# 2D exfoliated black phosphorus influences healthy and cancer prostate cell behaviors

**DOI:** 10.1038/s41598-021-85310-6

**Published:** 2021-03-12

**Authors:** Ines Fasolino, Alessandra Soriente, Maria Caporali, Manuel Serrano-Ruiz, Maurizio Peruzzini, Luigi Ambrosio, Maria Grazia Raucci

**Affiliations:** 1 Institute of Polymers, Composites and Biomaterials – National Research Council (IPCB-CNR), Mostra d’Oltremare pad.20 - Viale J.F. Kennedy 54, 80125 Naples, Italy; 2 Institute of Chemistry of Organometallic Compounds – National Research Council (ICCOM-CNR), Via Madonna del Piano 10, 50019 Sesto Fiorentino, Italy

**Keywords:** Cancer, Cell biology, Health care, Materials science

## Abstract

Nowadays, prostate cancer is the most widespread tumour in worldwide male population. Actually, brachytherapy is the most advanced radiotherapy strategy for the local treatment of prostate cancer. It consists in the placing of radioactive sources closed to the tumour side thus killing cancer cells. However, brachytherapy causes the same adverse effects of external-beam radiotherapy. Therefore, alternative treatment approaches are required for enhancing radiotherapy effectiveness and reducing toxic symptoms. Nanostructured exfoliated black phosphorus (2D BP) may represent a strategic tool for local cancer therapy because of its capability to induce singlet oxygen production and act as photosensitizer. Hence, we investigated 2D BP in vitro effect on healthy and cancer prostate cell behavior. 2D BP was obtained through liquid exfoliation. 2D BP effect on healthy and cancer prostate cell behaviors was analyzed by investigating cell viability, oxidative stress and inflammatory marker expression. 2D BP inhibited prostate cancer cell survival, meanwhile promoted healthy prostate cell survival in vitro by modulating oxidative stress and immune response with and without near-infrared light (NIR)-irradiation. Nanostructured 2D BP is able to inhibit in vitro prostate cancer cells survival and preserve healthy prostate cell vitality through the control of oxidative stress and immune response, respectively.

## Introduction

Recent high incidence rates of prostate adenocarcinoma are responsible of 20% of cancer-related deaths in the male Western population^1^. Prostate cancer is induced by the translocation of complex made by androgen receptor (AR) and his ligand [e.g. dihydrotestosterone (DHT) and testosterone, or other androgenic steroids] from the cytoplasm to the nucleus of prostate cells. After the translocation in the nucleus, AR-ligand-complex activates the transcription of numerous epigenetic factors and co-regulator proteins thus stimulating gene expression and the repression of oncosoppressor activity through post-translational modifications (phosphorylation, acetylation and ubiquitylation further fine-tune AR function)^2^. Numerous studies demonstrated that 90% of cases of prostate adenocarcinoma are organ-confined and it is possible to apply a local radiotherapy or prostatectomy^3^. Additionally, because of its hormone-responsivity the chemical androgen deprivation represents another therapeutic approach. However, the patients often become resistant to the hormone-therapy that shows a transient effectiveness (18–36 months). Another current strategy to treat prostate cancer is the use of AR competitive antagonists alone or in combination with anti-metastatic drugs or immunotherapy. This drug combination is useful for the treatment of not organ-confined prostate adenocarcinoma that often metastasizes in the bones^4^. Among the therapeutic approaches for the treatment of unresectable localized prostate carcinoma there is the low (LDR) and high dose rate (HDR) brachytherapy^5^. Brachytherapy belongs to radiation therapy strategies that are recommended for the curative treatment of male patients with prostate cancer. It consists on the transient or permanent implantation of radioactive sources into or very near target tissues. Indeed, “brachy” from the Greek term “brakhus” means “short” which refers to radiation therapy where radioactive sources are delivered really closed to cancer tissue^6^. Brachytherapy is useful for the treatment not only of prostate cancer but also of several malignancies such as cervical, uterine, breast, ocular, and skin cancers. Low dose rate brachytherapy commonly is obtained by placing permanently radioactive sources. This method differs from high dose rate (HDR) brachytherapy, where stronger radioactive sources are placed temporarily into the prostate and removed after the delivery of the effective dose^7^. From a medical standpoint, LDR brachytherapy represents a minimal-invasive procedure for the treatment of prostate cancer due to an accurate implantation of radioactive sources in a specific anatomical location. In addition, from a radiobiological point of view, the controlled dose escalation provided by LDR brachytherapy results more effective in killing tumour cells compared to conventional radiotherapy thus reducing the toxicity risks related to external beam radiation therapy (EBRT) that affect the bladder and rectum. Hence, LDR allows increasing EBTR dose about two times thus improving radiation effectiveness with a lower toxicity^8–10^. However, LDR causes some irreversible side effects but less troublesome compared to EBRT due to the radiation sources implantation. Some side effects arise after several weeks and may last for longer. These collateral symptoms include erection problems, inhibition of ejaculation, infertility, bowel problems and obstacles in urine passing with pain^11^.

In the field of regenerative medicine, much effort has been devoted to novel smart biomaterials with photo-thermal and photodynamic properties useful for cancer therapy that can substitute the conventional local radiotherapy^12^. In recent years, numerous studies support the use of Photodynamic Therapy (PDT) as minimally invasive curative approach with a selective cytotoxic activity toward cancer cells^13,14^. In this context, a relatively new member of the bi-dimensional family, exfoliated black phosphorus (2D BP) has been largely applied thanks also to the great advantage of being in vitro and in vivo biocompatible and biodegradable^15^, with a lower cytotoxicity, high mechanical properties, optical properties and topological features than other 2D materials [i.e. molybdenum disulphide (MoS_2_), hexagonal boron nitride (h-BN)], as the more spread graphene^16^. 2D BP is merely formed by P atoms connected by covalent single bonds, resulting in a hydrophobic surface able to interact for instance, with fatty acid chains of membrane lipids. At the same time, the lone pair of electrons on each P atom can be involved in additional hydrogen bonding or electrostatic interactions. The characteristic puckered structure of 2D BP^17,18^, ends in a high surface-to-area ratio which promotes its overall interaction with biomolecules and cells. Moreover, 2D BP has shown a good effectiveness as photodynamic therapy agent for cancer treatment because of its capability to produce singlet oxygen and its ability to act as photosensitizer^19^. Hence, in presence of reactive oxygen species (ROS) and infrared light irradiation, 2D BP represents an effective tool of PDT. However, in a previous study on osteosarcoma in vitro model we demonstrated that 2D BP possess intrinsic antiproliferative and anti-inflammatory properties that are independent of near infrared light (NIR)-irradiation^20^. According to this previous study, here we studied the effect of thermo-irradiated and not irradiated 2D BP on prostate cancer cells. At the same time, we have investigated how 2D BP influences healthy prostate cell behavior in view to propose 2D BP as a possible alternative to brachytherapy thus overcoming its drawbacks in terms of side effects on healthy tissue surrounding the tumor. For this purpose, we have studied the antiproliferative potential of 2D BP comparing cancer (PC-3 cell line) and healthy (PNT-2 cell line) prostate cell biological response. For better mimicking in vitro prostate cancer model, PC-3 cell line was chosen because it belongs to the triad (PC-3, LNCaP and DU-145 cells) that constitutes the gold standard of prostate cancer cell lines. Indeed, PC-3 cell line is similar to DU-145 cells, thus being hormone insensitive and showing no AR or PSA (prostate specific antigen) mRNA/protein, in order to reproduce prostate cancer conditions of patients that become resistant to the hormone-therapy^21^. Furthermore, PC-3 cell line is also used on in vivo model of prostate cancer as tool to track metastasis diffusion through intracardiac inoculation of genetically altered PC-3M-luc-C6 cells^21^. However, the main goal in cancer therapy is to protect healthy tissue surrounding tumor microenviroment. For this reason, here, the effect of 2D BP was also assessed on PNT-2 cell line. Indeed, PNT-2 cells are benign prostate epithelial cell line widely used to study the differences in protein expression profiles between benign and malignant prostate conditions for a better prostate cancer diagnosis and prognosis^22^.

## Methods

### Synthesis and characterization of 2D BP

**Figure 1:**
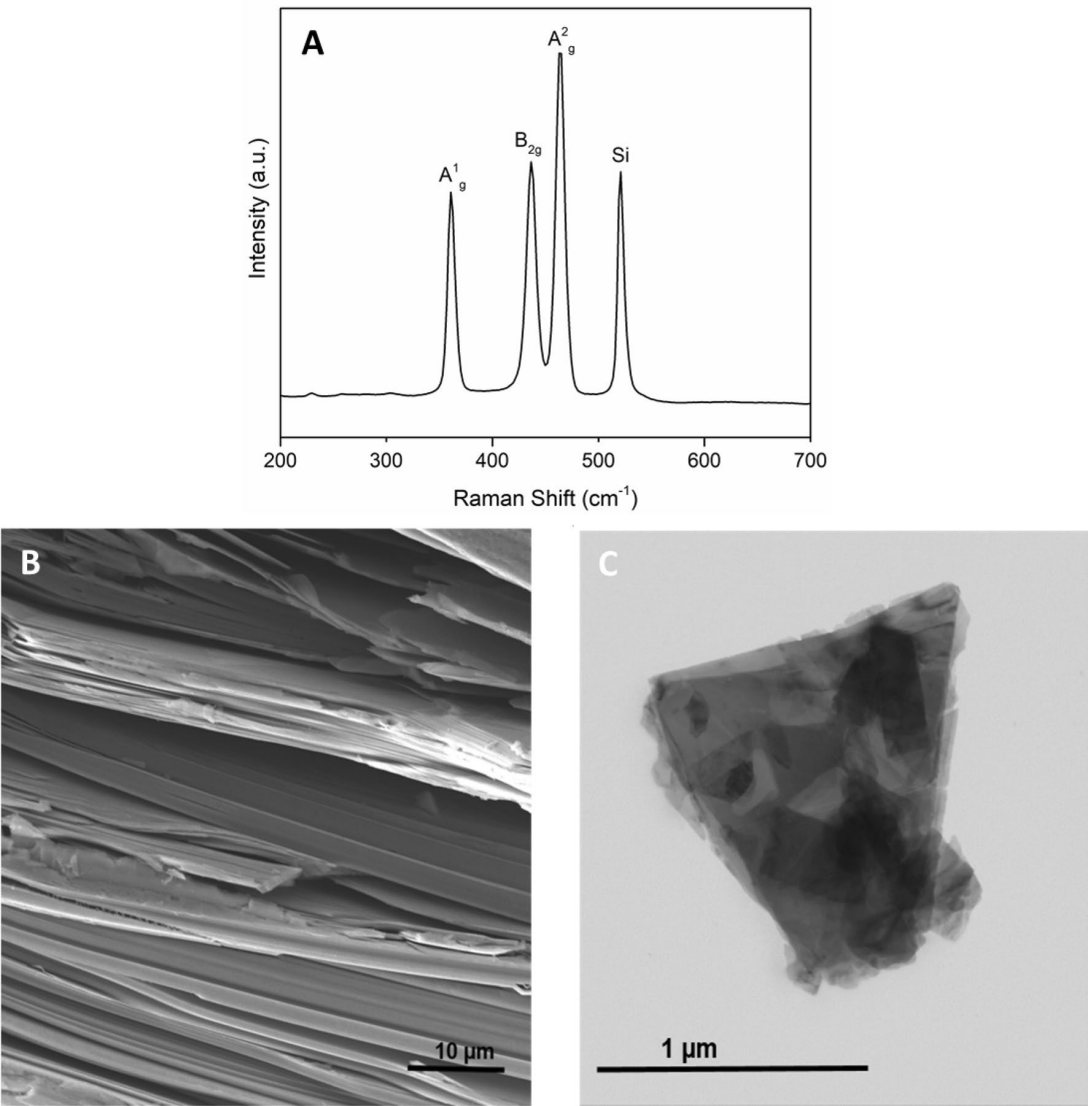
(**A**) Raman spectrum of 2D BP; (**B**) SEM image of BP microcrystals, scale bar: 10 µm; (**C**) Bright field STEM image of exfoliated BP nanosheets, scale bar: 1 µm.

Biological investigations were performed on 2D BP obtained by liquid exfoliation of bulk BP prepared according to literature^17,18^. High purity red phosphorus (99.99%) tin (> 99.999%), gold (> 99.99%) and a catalytic amount of SnI_4_ are put in a quartz vial that is evacuated and sealed under vacuum. The vial is heated in a muffle oven at T = 650 °C for three days and then cooled slowly to get high quality crystals. The purity of the material was ascertained by ICP-AES analysis, the morphological characterization of BP microcrystals was carried out by SEM, see Fig. 1B. The liquid phase exfoliation was performed by suspending bP microcrystals (5.0 mg, 0.161 mmol) in 5.0 mL of dry dimethylsulfoxide (DMSO) and then deoxygenated water (3.0–5.0 μL, 0.167–0.278 mmol) was added in order to get a molar ratio P/H_2_O = 1 – 0.6. The mixture was deoxygenated by vacuum and nitrogen, the vial was sealed and sonicated (37 MHz) for 5 days at 19 °C in the darkness. Afterwards, degassed ethanol (10.0 mL) was added and the suspension was centrifuged for 30 min at 9500 rpm. The supernatant was removed, and the treatment was repeated twice. The resulting dark grey powder was re-suspended by sonication (approximately one minute) in deionized water (1 mg/mL) and 1% of DMSO; the suspension was diluted in distilled water until to obtain a solution at 0.1% of DMSO that is not toxic for the cells. Later, the desired amount of this suspension was drop-casted on the well plate and left slowly to evaporate the water. To verify the morphology, purity and crystallinity of exfoliated black phosphorus, Scanning Transmission Electron Microscope (STEM), Raman spectroscopy and EDS analysis were performed.

### 2D BP in vitro effect on healthy and cancer cell lines

#### Cell cultures

Biological investigations were performed on 2D BP obtained by liquid exfoliation of bulk BP as detailed in previous “Synthesis and characterization of 2D BP” section. The experiments were performed using PNT-2 (human Normal prostate epithelium immortalized with SV40) and PC-3 (human Caucasian prostate adenocarcinoma) cell lines purchased from Sigma-Aldrich (Milano, Italy). Cells were cultured in 75 cm^2^ cell culture flask in RPMI 1640 and Dulbecco’s Modified Eagle’s Medium (DMEM) respectively supplemented with 10% Fetal Bovine Serum (FBS), antibiotic solution (streptomycin 100 µg/ml and penicillin 100U/ml, Sigma Chem. Co) and 2 mM L-glutamine^23^. PNT-2 cells at passage 9–11 and PC-3 cells at passage 11–15 were used for all the experimental tests and were grown at 37 °C in an incubator containing humidified atmosphere with 5% CO_2_ and 95% air.

#### 2D BP and near-infrared light irradiation (NIR) effect: proliferation, morphology and oxidative stress (nitrites, ROS, GPX-3, Iron levels)

Here, we have evaluated the behavior of 2D BP comparing PNT-2 and PC-3 biological response stimulated and not stimulated by NIR-irradiation. PNT-2 and PC-3 cells were seeded onto 2D BP at density of 1 × 10^4^ and cell behavior in terms of cell proliferation and ROS production was analyzed after two NIR-irradiation cycles of 15 min each. In particular, after 24 h of cell-material interaction, the cells were irradiated for 15 min as first time and then incubated overnight at 37 °C. Later, the cells were irradiated for a second time and cell proliferation was tested by Alamar Blue assay. Cells in plate without coating were used as control. After 24 and 72 h of seeding onto 2D BP (5–75 μg/mL), with and without NIR-irradiation, Alamar Blue reagent was incubated in each well. Optical density was measured with a UV–VIS spectrophotometer (Victor X3 Multilabel Plate Reader, Perkin Elmer) at wavelengths of 570 and 600 nm after 4 h of incubation at 37 °C that is time necessary for the cells to convert resazurin to resorufin. The optical density detected is directly proportional to metabolic activity of live cells. Furthermore, in order to study morphological changes, immunofluorescence analysis was also performed. Immunofluorescence analysis was carried out using a confocal laser scanning microscopy (Leica TCS sp8 confocal microscope). For this purpose, the cells were cultured in 8-well culture chamber at a density of 2 × 10^4^ cells/slides. After 48 h of exposure to 2D BP, in absence of NIR-irradiation, cells were fixed in 4% formaldehyde at room temperature for 10–30 min, washed three times with PBS and permeabilized with 0.1% Triton X-100 in PBS for 1 h. Cells without 2D BP treatment were used as plate controls. Then, the cells were washed 2–3 times in PBS and phalloidin-FITC conjugated working solution was incubated for 1 h. After that, samples stained with phalloidin were washed 3 times with PBS and cell images were acquired with a confocal microscope. Later, ROS species production in cells seeded onto 2D BP (5 μg/mL) was monitored by using the fluorescent probe DCFH-DA that, in presence of ROS, is rapidly oxidized to highly fluorescent dichlorofluorescein (DCF). The fluorescence intensity detected is directly proportional to the amount of ROS produced intracellularly. In order to measure ROS production, cells were incubated for 1 h with 10 μM solution of DCFH-DA in Hanks’ balanced salt solution containing 1% FBS; after the incubation, cells were washed thrice with PBS and treated with the Fenton’s reagent (H_2_O_2_/Fe^2+^ 2 mM) for 3 h at 37 °C. The DCF fluorescence intensity was detected through a fluorescent microplate reader (Victor X3, PerkinElmer), with the excitation wavelength of 485 nm and the emission wavelength of 538 nm. As another marker of oxidative stress, stable metabolites of nitric oxide were detected using Griess assay. For Griess assay, cells were seeded in a 48-multiwell plate at density of 5 × 10^4^ cells and were exposed to 2D BP (5 μg/mL) for 24 h. Later, cells were treated with Lipopolysaccharide (phlogistic agent) at concentration of 1 µg/mL as positive control for 24 and 72 h. After 2D BP exposure with and without NIR-irradiation cycles, 100 µL of cell supernatant was transferred into a 96-well plate and incubated with an equal volume of Griess reagent. After 1 h of incubation at RT, the absorbance was measured at a wavelength of 550 nm. To deepen the effect of oxidative stress on PNT-2 and PC-3 morphology, after cell-material interaction for 72 h, qualitative analysis was performed using Rhodamine phalloidin staining analyzed at confocal microscope (Leica TCS sp8) after cell fixation in 4% formaldehyde overnight as previously reported for phalloidin-FITC conjugated method. Furthermore, in order to investigate action’s mechanism of antioxidant potential of 2D BP, GPX-3 expression and intracellular Iron amount were measured respectively using immunofluorescence and Red Prussiate assays. For GPX-3 expression analysis, PNT-2 and PC-3 were plated on 2D BP or on glass slides alone (flat surface control) at density of 2 × 10^4^ cells/slide and incubated for 24 h. After cell permeabilization, as previously performed for phalloidin detection, GPX-3 polyclonal antibody (Bioss) ALEXA FLUOR 488 conjugated (1:100 dilution) was incubated in each slide at 4 °C overnight. After three washes with PBS, cells were treated with DAPI (10 μg/mL). Finally, cells were washed other three times with PBS and observed at a confocal microscope. For Iron levels detection, Red Prussiate reagent was used to obtain a colorimetric Iron dosing reaction. A standard curve was built using different concentrations of Iron nitrate and a blank solution was prepared with 100 μL of distilled water, 100 μL of 5 N HCl and 100 μL of Red Prussiate reagent. For Iron detection, PNT-2 and PC-3 (5 × 10^5^/well) were seeded onto 2D BP without NIR-irradiation. Then, cells were trypsinized and incubated with 5 N HCl solution at 37 °C for 3 days. After this time, 100 μL of samples were collected and incubated with 100 μL of Red Prussiate reagent for 15 min. The optical density (OD) was recorded at 450 nm using a spectrophotometer (Victor X3 Multilabel Plate Reader, Perkin Elmer).

#### 2D BP effect on in vitro ROS induced-cell death in cancer cells (PC-3): p53 expression

To investigate in vitro cell death induction by 2D BP, p53 expression was measured in absence of NIR-irradiation using immunofluorescence analysis. To this end, PC-3 cells (2 × 10^4^ cells/slide) were seeded onto glass slides with and without 2D BP. After 72 h of cell-material interaction, samples were fixed in 4% formaldehyde overnight and qualitative analysis on p53 expression was performed using a confocal microscope (Leica TCS sp8). As previously described for phalloidin detection, monoclonal rabbit-p53 antibody (Invitrogen) (1:200 dilution) was incubated in each slide at 4 °C overnight. After three washes with PBS, cells were treated for 2 h with FITC conjugated secondary antibody (goat-anti rabbit) at dilution of 1:500. Later, cells were washed thrice with PBS and incubated with DAPI (10 μg/mL). Finally, cells were washed other three times with PBS and observed at a confocal microscope. Quantitative data were collected using image analysis through ImageJ software (ImageJ 1.44 which uses Java 1.6 in 64-bit mode). Mean of fluorescence intensity was quantified on a triplicate of each sample. A defined area within the image was selected and the pixel intensity object plus background at that pixel on the toolbar was read out.

#### 2D BP effect on in vitro healthy prostate cell (PNT-2) immune response

##### Enzyme-linked immunosorbent assay (ELISA) and Griess assay for pro-inflammatory and anti-inflammatory marker determination

Pro- and anti-inflammatory interleukins secretion after 2D BP (5 μg/mL) treatment (with and without NIR-irradiation) was measured by using commercial ELISA kits (Affimetrix Italia, SRL) according to the manufacturer’s instructions. After 24 h of seeding (1 × 10^5^ cells/well), phlogistic stimulus was obtained using LPS at concentration of 1 µg/mL. After 3 days, interleukin-10 (IL-10) and interleukin-6 (IL-6) levels in PNT-2 supernatants were quantified. Then, on the same supernatants, nitrites levels were also analyzed using Griess assay to confirm LPS stimulation after 72 h of exposure.

##### Cyclooxygenase-2 (COX-2) protein expression

To study the anti-inflammatory potential of 2D BP on healthy prostate cells (PNT-2) stimulated with LPS at concentration of 1 µg/mL for 72 h, COX-2 expression was detected through western blot analysis. Protein extracts were collected from PNT-2 cells seeded at density of 5 × 10^5^ onto 2D BP. The protein amount was determined using Bradford method and the proteins were subjected to electrophoresis on 10–15% polyacrylamide precast gel. Later, the proteins were electrophoretically transferred to a nitrocellulose membrane and blocked in 5% non-fat dry milk buffer for 1 h. After blocking, the membranes were incubated with rabbit anti-COX-2 (Bioss Antibodies) at 1:200 dilution overnight at 4 °C. Then, the membranes were washed and incubated with rabbit-anti-peroxidase-conjugated goat IgG (Abcam Antibodies) at 1:1000 dilution and the signal was detected after incubation of Clarity Max Western ECL Substrate (BIO-RAD) using Versa Doc Imaging System (BIO-RAD). The resulting bands were normalized on β-actin (Abcam Antibodies) and analyzed using Quantity One software version 4.6.3.

##### SOD (superoxide dismutase) activity

Superoxide radical scavenging capacity of 2D BP at day 3 of LPS exposure in PNT-2 was analyzed to confirm 2D BP anti-inflammatory and antioxidant effect in normal prostate tissues. Superoxide dismutase (SOD) assay was performed using the Total SOD assay Kit Elabscience (Hydroxylamine method) according to manufacturer’s instructions. Absorbance readings at 550 nm were recorded using a spectrophotometer (Victor X3 Multilabel Plate Reader, Perkin Elmer) and SOD activity was calculated as Inhibition ration = OD_control _− OD _sample_/OD_control_ * 100% and expressed as SOD U/mL.

#### Statistical analysis

Statistical analyses were undertaken using GraphPad Prism, version 5.00 (GraphPad Software, La Jolla California USA, www.graphpad). Data were compared using a Student's t-test, a one-way ANOVA, with a Bonferroni post-test (parametric methods). The results are expressed as Mean ± standard deviation (SD). Values of *p* < 0.05 were considered significant.

## Results and discussion

Prostate cancer represents one of the most malignant tumors affecting worldwide male population. The higher incidence and a not easily diagnosis make prostate cancer a big threat for male gender^24^. Hence, a lot of studies are aimed at identifying genes and signaling pathways to target for the regulation of cell proliferation and apoptosis in prostate cancer. To inhibit selectively cancer, cell viability is a crucial step for blocking tumorigenesis and metastasis processes.

### Purity and morphological characterization of 2D BP

In the present study, we assessed 2D BP effect on cell proliferation and cell death mechanisms in order to find a novel strategy based on higher performance biomaterials for prostate cancer treatment. To achieve this aim, pure exfoliated black phosphorus obtained by liquid exfoliation was used. The morphology and the purity of the 2D BP were examined by physico-chemical and morphological characterizations. In particular, Raman spectrum was registered and the three characteristic peaks of BP at 357.8, 431.5 and 459.2 cm^−1^ corresponding to the active Raman phonon modes of black phosphorus, A1g, B2g and A2g respectively were observed (Fig. 1A). The chemical purity was also assessed by EDS analysis, see Fig. S1. Morphological analysis of the synthesized 2D BP was carried out by STEM, see Fig. 1C, and showed few layers BP flakes, with lateral dimensions going roughly from 300 nm to 1.2 µm. Meanwhile, Fig. 1B is representative of the bulk BP.

### Effect of 2D BP on PNT-2 and PC-3 cell proliferation

**Figure 2:**
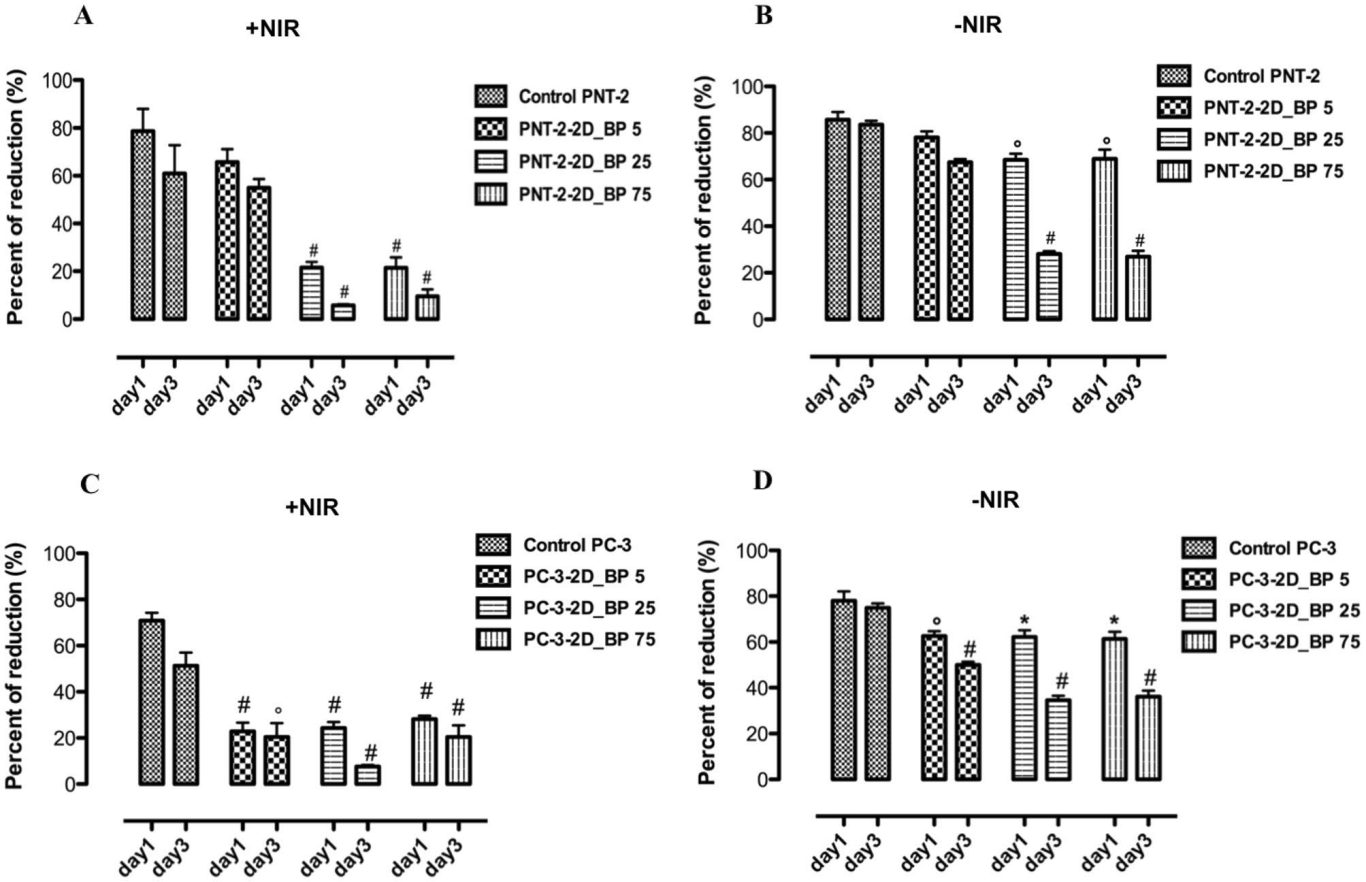
Effect of 2D BP on PNT-2 (**A**, **B**) and PC-3 (**C**, **D**) proliferation with and without infrared irradiation (NIR). The experiments performed on PNT-2 and PC-3 cell proliferation using Alamar Blue assay have shown that irradiated (**A**–**C**) and not irradiated (**B**–**D**) 2D BP (5 μg/mL) did not exert cytotoxic effects on PNT-2 and PC-3 after 1 and 3 days of exposure. The same result was obtained in presence of not irradiated 2D BP (25 μg/mL) at day 1 of cell culture (**A**). Not irradiated 2D BP (75 μg/mL) at day 3 and irradiated 2D BP (25–75 μg/mL) at all-time points significantly (°*p* ≤ 0.001; ^#^*p* ≤ 0.0001) decreased cell viability of healthy prostatic cells (PNT-2) compared to control (**A**, **B**). Results are mean ± SEM of 3–4 experiments. The irradiated (**C**) and not irradiated (**D**) lowest concentration (5 μg/mL) of 2D BP induced a significant (°*p* ≤ 0.001; ^#^*p* ≤ 0.0001) decrease in PC-3 (cancer cells) proliferation after 1 and 3 days of exposure compared to control. This effect results more significant in presence of irradiation (**A**). The best inhibition in PC-3 proliferation was obtained after 3 days of irradiated 2D BP exposure at concentration of 75 μg/mL ^#^*p* ≤ 0.0001 (**A**). Results are mean ± SEM of 3–4 experiments.

**Figure 3:**
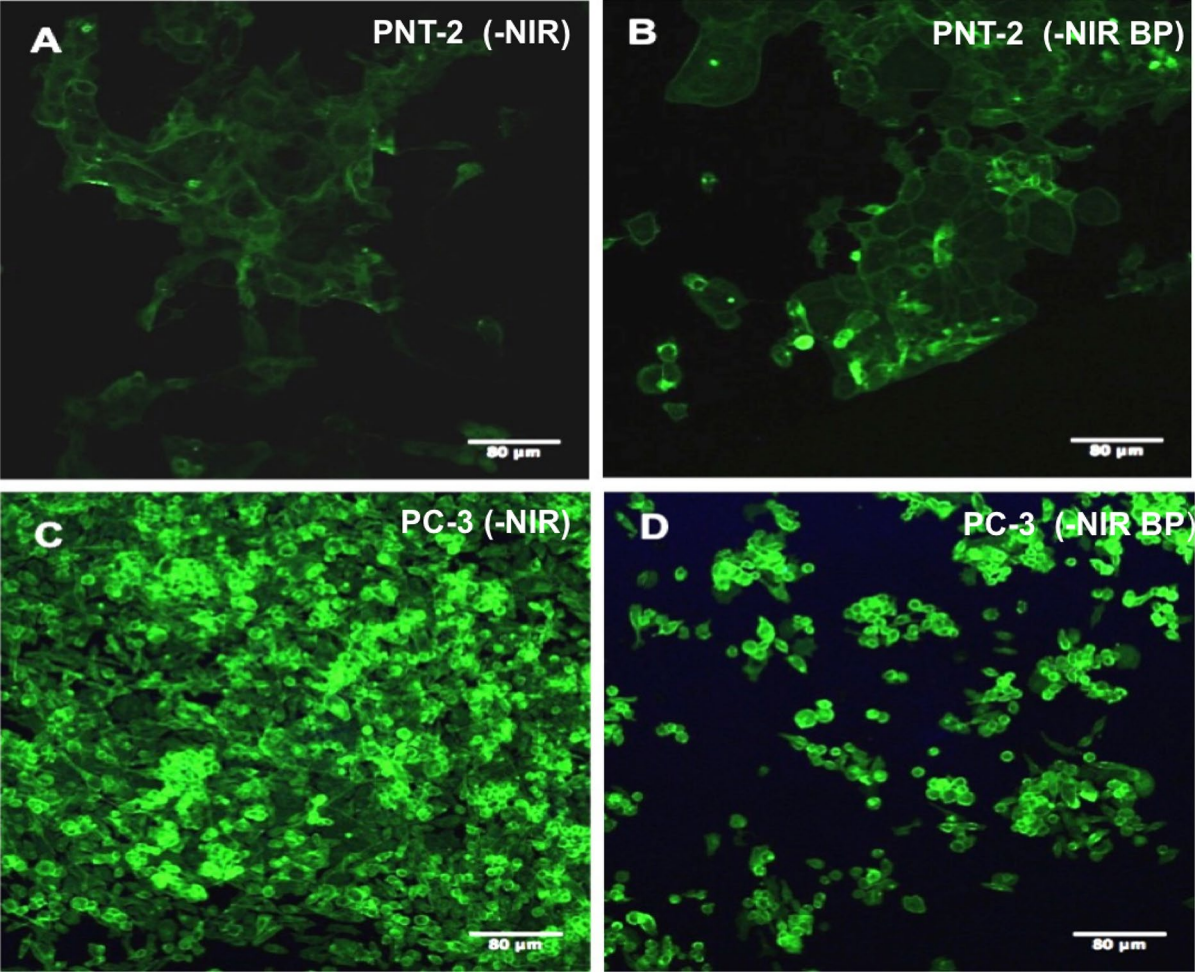
Effect of not irradiated (-NIR) 2D BP on PNT-2 and PC-3 cell density (qualitative analysis). PNT-2 and PC-3 cells grown in absence (**A**, **C**) and in presence (**B**, **D**) of 2D BP. The exposure of PNT-2 cells to 2D BP does not change cell density (**B**) compared to control (**A**). By contrasts, 24 h of 2D BP treatment reduces PC-3 density (**C**) compared to cells in the control (**D**). The images are representative of three experiments. Cell morphological features at 72 h 2D BP treatment are reported in Fig. S2 of Supporting information section.

To evaluate the anti-proliferative potential effect of 2D BP on cancer cells, cell survival was detected. To this end, cell proliferation was analyzed on PNT-2 (healthy) and PC-3 (cancer) cells in presence of 2D BP (5–75 μg/mL) with and without NIR-stimulation. The range of 2D BP concentrations 5–75 μg/mL was chosen in order to find the best concentration useful to kill cancer cells but not toxic for the surrounding healthy prostate cells. The experiments performed on PNT-2 and PC-3 cell proliferation using Alamar Blue assay have shown that irradiated and not irradiated 2D BP (5 μg/mL) did not exert cytotoxic effects on PNT-2 after 1 and 3 days of exposure (Fig. 2). The same result was obtained in presence of not irradiated 2D BP (25 μg/mL) at day 1 of cell culture. Conversely, not irradiated 2D BP (75 μg/mL) at day 3 and irradiated 2D BP (25–75 μg/mL) at all-time points significantly (°*p* ≤ 0.001; ^#^*p* ≤ 0.0001) decreased cell viability of healthy prostate cells (PNT-2) compared to control. Meanwhile, the irradiated and not irradiated lowest concentration (5 μg/mL) of 2D BP induced a significant (°*p* ≤ 0.001; ^#^*p* ≤ 0.0001) decrease in PC-3 (cancer cells) proliferation after 1 and 3 days of exposure compared to control. However, this effect results more significant in presence of NIR-irradiation. The best inhibition in PC-3 proliferation was obtained after 3 days of irradiated 2D BP exposure at concentration of 75 μg/mL (^#^*p* ≤ 0.0001) but this concentration is toxic for PNT-2 cells. For this reason, the majority of analyses were performed using the lowest concentration of 2D BP (5 μg/mL), without NIR-irradiation, that decreased in a significant manner PC-3 proliferation without exerting any cytotoxic effect on PNT-2 healthy cells. Qualitative analysis obtained through immunofluorescence assay confirmed results on cell survival. Indeed, in Fig. 3, PNT-2 after 24 h of 2D BP (B) exposure showed the density of the control (A). Conversely, a reduction in PC-3 cell density was observed after 24 h of 2D BP exposure (D) compared to control where cells appeared well spread and at higher density (C). In addition, cell morphological features are reported in Fig. S2. Figure S2A is representative of PNT-2 after the interaction of 72 h with 2D BP and a good cell was observed. Conversely, PC-3 cells showed a collapsed cell shape after 72 h of interaction with 2D BP without NIR-irradiation (Fig. S2B). Indeed, PC-3 cells have lost their specific tapered shape thus indicating cell-suffering condition. These results pave the way to the exploration of novel methods apt to induce cancer cell death preventing healthy cell impairment. Taking into account 2D BP effect on cell proliferation, it is pivotal to discover the mechanisms of action and the molecular pathway underlying to the selective cancer cell death. For this purpose, the following data discuss the potential involvement of oxidative stress and cell cycle arrest in cancer cell death.

### Effect of 2D BP on oxidative stress in PNT-2 and PC-3 cells

Recently, selective ROS induction in cancer cells has been paid much attention as strategy to inhibit cancer cell proliferation^25^. It is well known that cancer cells spontaneously produce high levels of reactive oxygen species deriving of an increased metabolic activity and a constant crosstalk with infiltrating immune cells^26^. The seminal work of Xie et al. discovered that BP nanosheets are efficient photosensitizers since they can generate singlet oxygen under the entire visible light region. This means 2D BP can promote the formation of ROS, whose excessive level is responsible of oxidative stress in cells, inducing lipid peroxidation, reduction in catalase activity and DNA breakage^19^.

**Figure 4:**
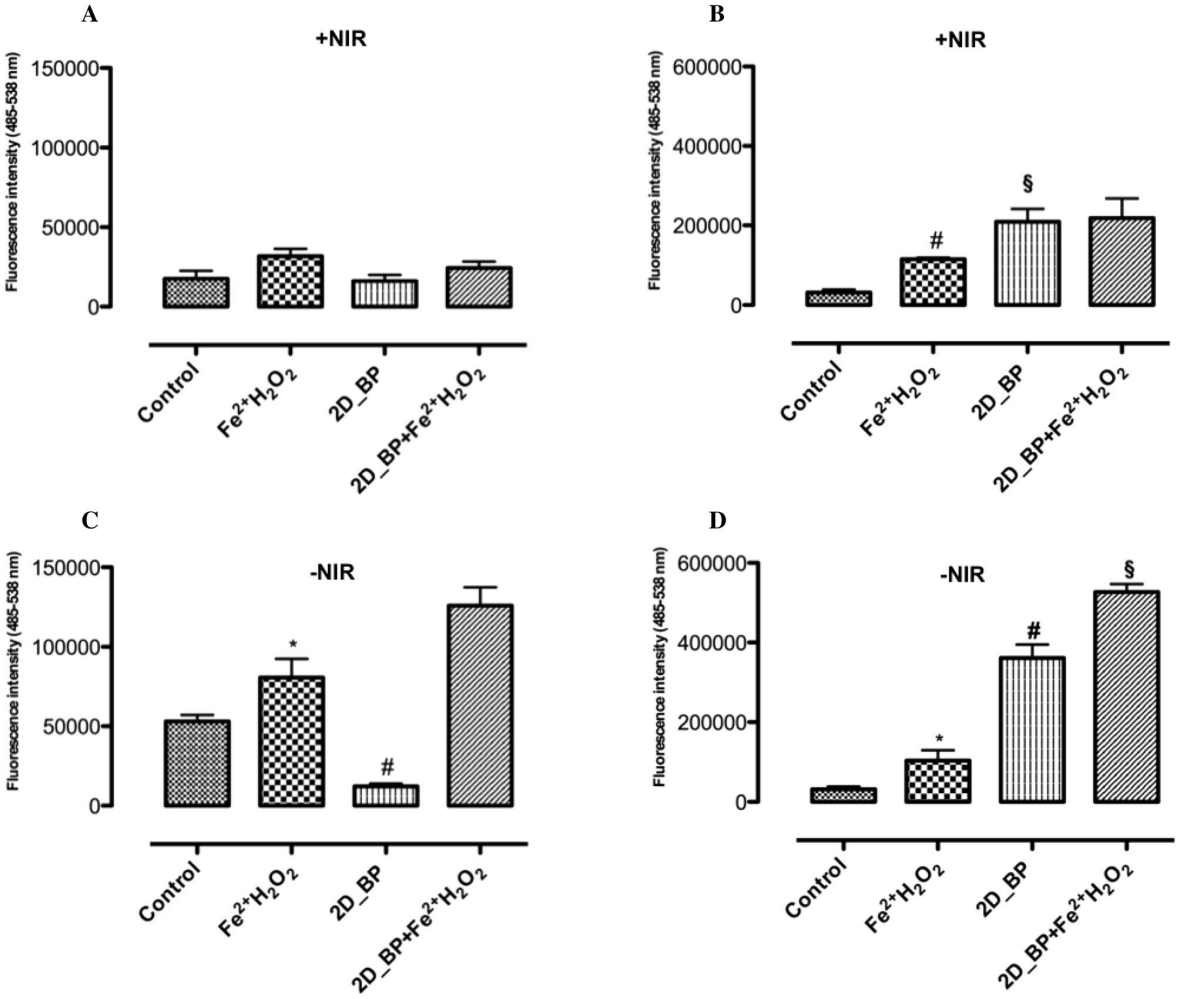
Reactive Oxygen Species (ROS) production from PNT-2 (**A**, **C**) and PC-3 (**B**, **D**) cells with (+ NIR) and without infrared (-NIR) irradiation. The exposure of PNT-2 and PC-3 cells (healthy and cancer cells) to H_2_O_2_/Fe^2+^ (2 mM) significantly (*p ≤ 0.05, ^#^ *p* ≤ 0.0001) increases ROS production compared to control (**A**–**D**). As irradiated 2D BP with H_2_O_2_/Fe^2+^ (2 mM) irradiated 2D BP without H_2_O_2_/Fe^2+^ (2 mM) stimulation was able to induce a significant (^#,§^p ≤ 0.0001) increase in ROS generation in PC-3 cells (**B**, **D**). Not irradiated 2D BP significantly (#*p* ≤ 0.0001) reduced basal ROS levels compared to control on PNT-2 cell line (**C**). Conversely, not irradiated 2D BP did not inhibit ROS generation induced by H_2_O_2_/Fe^2+^ (2 mM) in PNT-2 cells compared to H_2_O_2_/Fe^2+^ (2 mM) alone (**C**). Results are mean ± SEM of 3–4 experiments.

**Figure 5:**
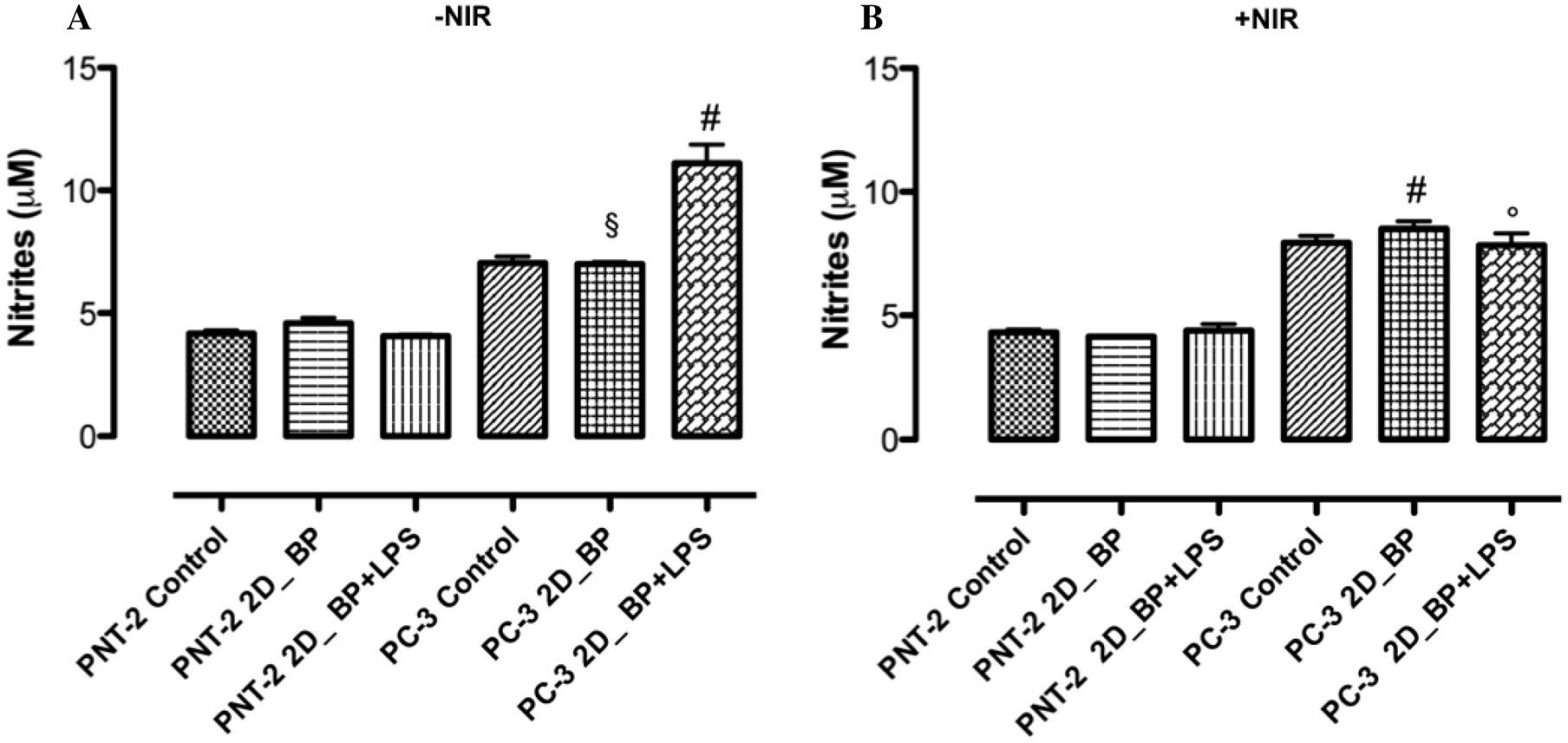
Nitrites production from PNT-2 and PC-3 cells without (**A**) and with (**B**) infrared irradiation (NIR). The results suggest that 48 h of exposure to 2D BP without and with irradiation significantly (^§^*p* ≤ 0.0001) increase basal nitrites values in PC-3 cells compared to PNT-2. This effect is more evident in presence of pathological conditions obtained through LPS (1 μg/ml) stimulation (^#^*p* ≤ 0.0001) and irradiation (^#^,°*p* ≤ 0.0001), (**B**). Results are mean ± SEM of 3–4 experiments.

Starting from that, we investigated 2D BP capability to increase ROS production in PC-3 prostate cancer cells compared to healthy PNT-2 cells in presence and in absence of NIR-irradiation. The results demonstrated that the exposure of PNT-2 (healthy cells) to H_2_O_2_/Fe^2+^ at concentration of 2 mM (oxidative agent) induced an increase in ROS production (Fig. 4A). Irradiated 2D BP (5 μg/mL) did not significantly change basal ROS levels compared to control (Fig. 4A). However, a pre-treatment with 2D BP (2D BP H_2_O_2_/Fe^2+^) for 24 h reduced ROS generation induced by H_2_O_2_/Fe^2+^ (2 mM) compared to H_2_O_2_/Fe^2+^ (2 mM) alone (Fig. 4A). However, in the case of PC-3 cells, stimulation with H_2_O_2_/Fe^2+^ (2 mM) induced a significant (^#^*p* ≤ 0.0001) increase in ROS production. Furthermore, irradiated 2D BP with and without H_2_O_2_/Fe^2+^ (2 mM) stimulation induced a significant increase in ROS generation thus determining a permanent damage in cancer cells (Fig. 4B). The exposure of PNT-2 and PC-3 cells (healthy and cancer cells) to H_2_O_2_/Fe^2+^ (2 mM) significantly (*p ≤ 0.05) increases ROS production compared to control. Not irradiated 2D BP significantly (^#^*p* ≤ 0.0001) reduced basal ROS levels compared to control on PNT-2 cell line (Fig. 4C). Conversely, not irradiated 2D BP did not inhibit ROS generation induced by H_2_O_2_/Fe^2+^ (2 mM) in PNT-2 cells compared to H_2_O_2_/Fe^2+^ (2 mM) alone (Fig. 4C). On cancer cells (PC-3), not irradiated 2D BP with and without H_2_O_2_/Fe^2+^ (2 mM) stimulation induced a significant (^#^’^§^*p* ≤ 0.0001) increase in ROS production thus suggesting 2D BP ability of causing oxidative stress in cancer prostate cell lines (Fig. 4D). Indeed, several previous studies have demonstrated an increasing of hydrogen peroxide and nitrite oxide levels in tumour cells in response to inflammatory stimuli such interferon γ (IFNγ), tumor necrosis factor-alpha (TNFα) and LPS thus confirming a higher crosstalk with immune system. Additionally, many recent findings have shown that immune response plays a pivotal role in cancer progression and ROS induced by neutrophils and macrophages contribute to kill tumour cells^26^. It was also established that, during inflammatory response, immune cells such as macrophages also generate nitric oxide which, combined to superoxide, generates to peroxinitrite radicals that contribute to induce tumour cell death^27^. The literature reported that 2D BP is considered as graphene (GO) and graphene-analogous 2D nanosheets with important properties for biomedical applications^28^. In particular, several studies reported the ability of graphene to interact with immune system as tested on in vitro and in vivo experimental models. Indeed, biomedical and pharmaceutical research reports that GO interacts with human carcinoma cervical cells (HeLa) in vitro by activating immune system and cancer cells^29^. Furthermore, GO as 2D system causes specific interactions with blood proteins and biological membranes leading to in vivo immune cell response activity^30^. Hence, firstly, we have evaluated the biological response also in terms of nitrite oxide production. To this end, we simulated an inflammatory condition stimulating PNT-2 and PC-3 with LPS in presence of irradiated and not irradiated 2D BP for 24 h. The results demonstrated that 2D BP with and without NIR-irradiation (Fig. 5 A, B) was able to decrease nitrite production in healthy PNT-2 cells (Fig. 5A). An opposite behavior was observed in PC-3 cancer cells, where 2D BP without irradiation maintained high nitrites levels induced by LPS (Fig. 5A). This effect is more significant in presence of NIR-irradiation as showed in Fig. 5B. Indeed, irradiated 2D BP was able to increase also basal levels of nitrite without LPS stimulation. The effect of 2D BP on nitric oxide generation confirmed the potential anticancer activity related to a possible saturation of radical species responsible of cell death induction in cancer tissues.

**Figure 6:**
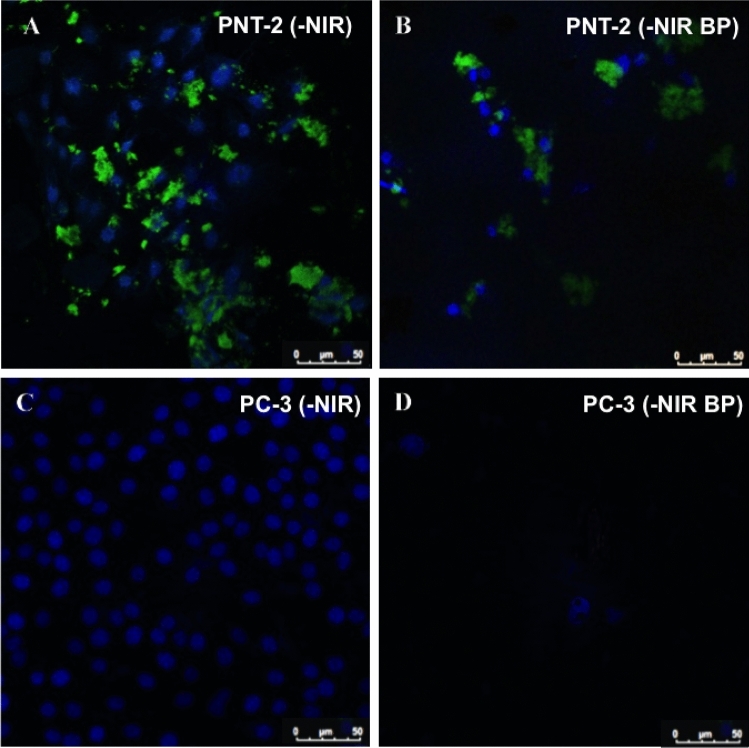
GPX-3 expression in PNT-2 (**A**, **B**) and PC-3 (**C**, **D**) cells in presence of 2D BP without infrared (-NIR) irradiation. PNT-2 and PC-3 cell GPX-3 expression in absence (A,C) and in presence 2D BP exposure at concentrations of 5 (B,D) μg/mL after of 24 h of cell culture. The images are representative of three experiments.

In order to discover the pathway involved in 2D BP mechanism of action, here the intracellular Iron amount was measured on the basis of previous studies that have showed an excess of radical species induced by Iron overload with the saturation of the buffer mechanisms thus inducing a dramatic increasing of oxidative stress^31^. The induction of cytotoxicity in PC-3 was confirmed by the increase of Iron levels (Fig. S3) and the oxidative stress after cell-material interaction. In addition, 2D BP did not change basal values of Iron in PNT-2 thus suggesting the ability of 2D BP to protect buffer mechanisms (antioxidant activity) in physiological conditions. Glutathione peroxidase (GPX) enzyme-family (GPX1–3 and 4) plays a pivotal role in the protection of buffer mechanisms reducing hydrogen peroxide (H_2_O_2_) and lipid hydroperoxides levels. In this way, GPX enzymes play a crucial role in cell detoxification thus inhibiting cellular damage induced by ROS^32^. To confirm the effect of 2D BP on oxidative stress, GPX-3 expression was measured in PNT-2 and PC-3 cells after 24 h of 2D BP exposure using immunofluorescence analysis. As reported in Fig. 6A, PNT-2 cells seeded on plate surface expressed basal levels of GPX-3 after 24 h of cell culture. 2D BP exposure for 24 h allowed to store antioxidant enzymes activity expressed as GPX-3 green signal in PNT-2 cells (Fig. 6B). By contrast, in PC-3 cells that did not express GPX-3 in basal condition (Fig. 6C), 24 h of 2D BP exposure did not activate buffer mechanism in terms of GPX-3 green signal (Fig. 6D). Furthermore, in Fig. 6D, PC-3 cell density was significantly reduced after 24 h of cell-material interaction compared to plate control (Fig. 6C). This latter result is in line with the previous effects of 2D BP on cell proliferation and ROS generation.

### Effect of 2D BP on ROS-induced p53 expression in cancer cells (PC-3) as cell death marker

In the field of scientific subjects concerning the healthcare, the therapy of malignant tumors covers a key role considering the high incidence of deaths related to aggressive and often untreatable forms of cancer^33^. In this context, the selective induction of cell death in cancer cells is becoming one of the major challenges to rise. Indeed, recent clinical trials confirmed that the induction of cell death enhances the effectiveness of standard anticancer drugs^34^. In order to deepen 2D BP mechanism of action, here we hypothesize the connection between ROS increase and cell death induction. Indeed, recently it has been discovered that the overexpression of intracellular ROS following peroxide treatment caused cell death of cancer cells under oxidative stress conditions^35,36^. In this study, to confirm the activation of cell death mechanisms following higher ROS levels induced by 2D BP in cancer cells (PC-3), p53 gene expression was investigated through confocal analysis. A main feature of cancer cells is the deregulation of cell cycle control, where several genes such as p53 are involved. Indeed, it was widely reported that the suppression of the p53 gene determines an uncontrolled proliferation of cancer cells. Conversely, the expression of p53 causes a stable cell growth arrest or programmed cell death by encoding for proteins with physiological and biological properties. In more detail, additional biochemical and pharmacological studies revealed that p53 induces cell death through a three-phases: (1) transcription of redox-related genes; (2) the induction of reactive oxygen species generation and (3) the degradation of mitochondrial components induced by oxidative conditions, resulting in cell death^37^.

**Figure 7:**
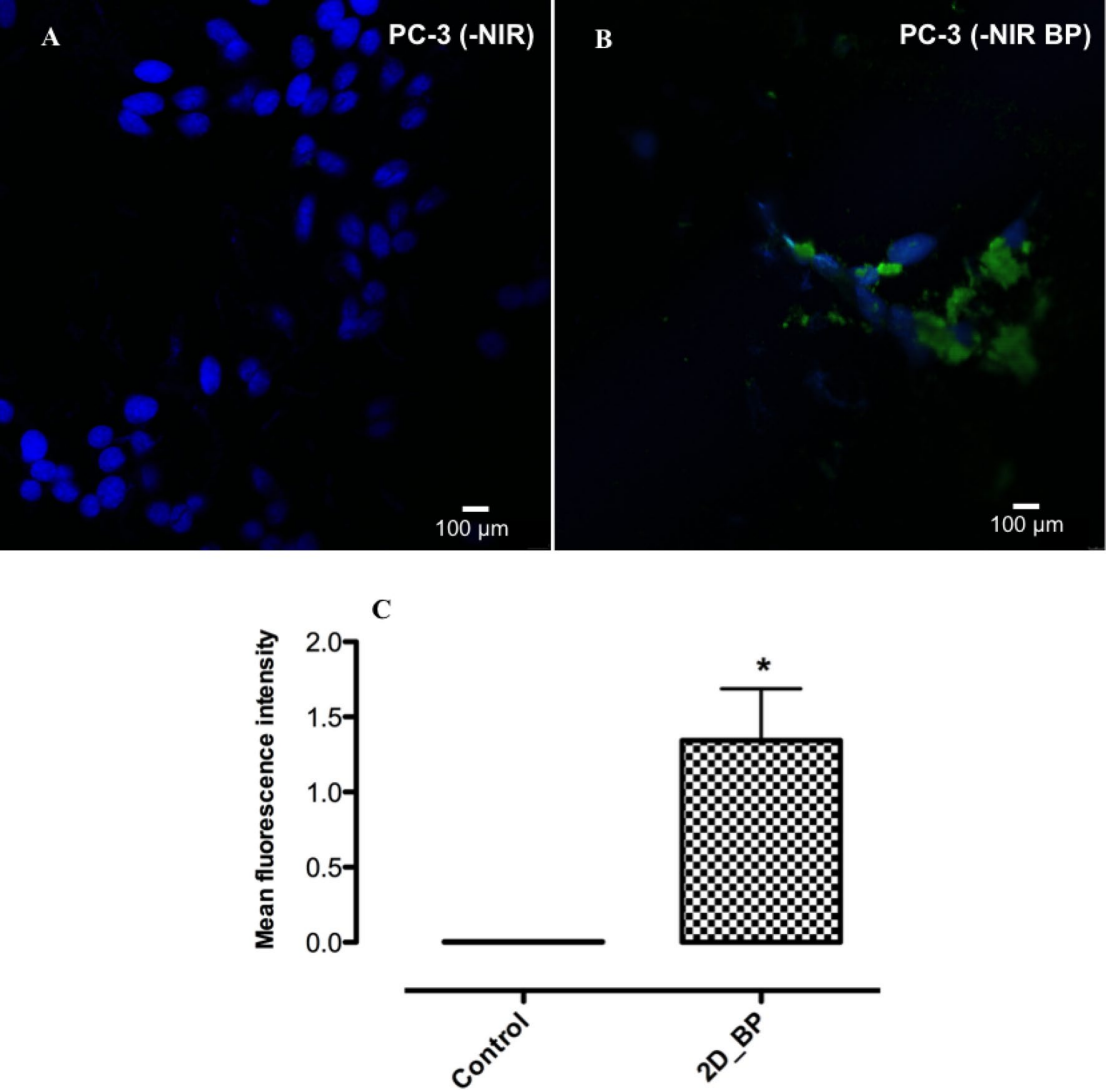
Effect of not irradied (-NIR) 2D BP on qualitative p53 expression in PC-3 cells (immunofluorescence). P53 expression in PC-3 cells in absence (**A**) and in presence (**B**) of 2D BP exposure (5 μg/mL) for 72 h. The images are representative of three experiments. The fluorescence intensity was expressed as mean of fluorescence intensity ± SD (*p ≤ 0.05 vs Control = PC-3 cells without 2D BP treatment).

Figure 7A, B show p53 expression in PC-3 cells exposed or not to 2D BP treatment. Indeed, 72 h of exposure to 2D BP (5 μg/mL) significantly induced p53 expression marked by green signal compared to control (PC-3 not exposed to 2D BP), where it is possible to appreciate only DAPI (nuclei) related signal. Qualitative data on p53 expression were confirmed by image analysis showed in Fig. 7C. These results are relevant in view to discover alternative technologies to External beam radiation therapy for localized prostate cancer treatment. Specifically, it was established that External beam radiation therapy is an effective tool for treating localized prostate cancer, although failures occur at high rates. However, it was previously discussed that cancer cells express genes such as Slug that are able to inhibit p53-mediated cell death and is often up-regulated after irradiation of radioresistant cancers^38^. Recently, activation of the mitochondrial (intrinsic) pathway of apoptosis by ROS through the exogenous or endogenous induction of p53 plays a pivotal role in the control of cellular stress responses, including cell cycle arrest for promoting DNA repair or cell death^35^. Our findings suggest that the potential use of 2D BP in combination or in replacement of External beam radiation therapy could allow to treat locally also radioresistant prostate cancer forms by increasing the effectivness of mechanism of control of cell-cycle and cell death mediated by p53 suppressor gene induced by ROS at higher doses. Hence, 2D BP capability to exert toxic effects on cancer cells may constitute a technological advancement in the field of brachytherapy and it could be replaced to radiation sources.

### Effect of 2D BP on in vitro inflammatory response in healthy prostate (PNT-2) cells

Inflammatory mediators and immune cells are the main residents in local cancer microenvironment. Many cancer types are the result of previous inflammatory conditions. By contrast, in other cancer types the inflammation occurs as consequence of the tumour development. Indeed, it is well known that cancer cells are able to modify the inflammatory response in order to create an environment that promotes cancer growth^39^. Regarding chronic inflammatory diseases, the high correlation between chronic inflammation and tumour development was widely discussed. Hence, there is a need to prevent tumour-promoting effects by blocking chronic disorders progression. Furthermore, the identification of not jet-disclosed cancer-related inflammatory molecular pathway could improve cancer prevention, diagnosis and treatment. To evaluate 2D BP anti-inflammatory potential on prostate healthy cells we measured inflammatory mediators (nitric oxide, IL-10, IL-6 and COX-2 levels) produced by cells after LPS (1 μg/mL) stimulation for 3 days for better mimicking the phlogistic microenvironment. Hence, LPS (1 μg/mL) induced high levels of nitric oxide in PNT-2 after 3 days of stimulation compared to control (Fig. S4A, B). Irradiated and not irradiated 2D BP without LPS did not change basal levels of nitrites in PNT-2 cells (Fig. S4A, B). By contrast, 2D BP significantly reduced nitric oxide production induced by LPS levels. These results are in agreement with the ability of 2D BP to inhibit the activation of pathway involved in oxidative stress (index of inflammation) in physiological in vitro conditions as previously discussed. Furthermore, 2D BP effects in physiological conditions (healthy prostate cells) were studied by measuring several pro- and anti-inflammatory interleukins involved in the immune response. The results suggested that irradiated and not irradiated 2D BP stimulated the production of anti-inflammatory cytokines such as IL-10 (Fig. S4C, D) and in parallel was able to significantly inhibit pro-inflammatory cytokines (IL-6) production but only without irradiation (Fig. S4E, F). Additionally, COX-2 expression, that is the most important target for conventional non-steroidal anti-inflammatory drugs useful for the treatment of inflammation and pain, was also detected. As showed in Figure S4G, 2D BP inhibited COX-2 expression in PNT-2 cells stimulated by LPS. As last step, we explored SOD activity. SOD is an enzyme involved in specific inhibition of superoxide anion free radical (O_2_^−^) thus reducing the formation of nitrite and inflammatory cascade activation^40^. Indeed, SOD plays a key role as antioxidant defence in the pathogenesis of several inflammatory diseases by interfering with ROS generation, which determines tissue destruction^41^. Results on SOD activity demonstrated that 3 days of LPS exposure produced a significant decrease in SOD activity compared to control without phlogistic stimulus, meanwhile 2D BP exposure counteracted LPS-induced reduction in SOD activity. Moreover, 2D BP without LPS stimulation significantly induced basal SOD activity (Fig. S5). In addition, a consistent number of studies showed the involvement of ROS in DNA damage processes, thus causing mutagenesis and genotoxic effects^42^. In the present study, we have observed a significant increase in SOD activity (index of good buffer mechanisms functionality) in PNT-2 cells treated with 2D BP in basal conditions (without LPS stimulation). More importantly, after LPS stimulation, which activates oxidative stress and several signalling transduction pathways [extracellular-signal-related kinases (ERK), mitogen-activated protein kinase (MAPK) family, and nuclear factor kB (NF-kB)], 2D BP has shown to preserve physiological SOD activity, thus suggesting the opportunity to control the DNA damage induced by ROS production.

## Conclusions

Over the past decades, Brachytherapy has been proven a highly effective approach for the treatment of localized unresectable prostate cancer in terms of safety and cost-effectiveness. Nonetheless, it causes some permanent symptoms as external-beam radiotherapy due to the radiation sources implantation. Considering the imperative need of novel strategies for prostate cancer treatment, here we propose the in vitro study of a non-radioactive alternative based on nanostructured exfoliated black phosphorus on healthy and cancer cell behavior. This 2D BP has previously shown the ability to inhibit in vitro bone cancer and simultaneously promote normal bone cell survival^20^. In the present work, 2D BP was capable to inhibit in vitro prostate cancer cell survival and protect healthy prostate cells through the control of oxidative stress and inflammation, respectively.

## Supplementary Information

Supplementary Figures.
