# Supplementary material for: 2D exfoliated black phosphorus influences healthy and cancer prostate cell behaviors
Source: Sci Rep. 2021 Mar 12;11:5856. doi: 10.1038/s41598-021-85310-6 (PMC7955096; doi:10.1038/s41598-021-85310-6)
Supplement: Supplementary file 1 — Supplementary Figures. [file 41598_2021_85310_MOESM1_ESM.doc]

**2D Exfoliated Black Phosphorus influences Healthy and Cancer Prostate Cell behaviors**

**Ines Fasolino^1*^, Alessandra Soriente^1^, Maria Caporali^2^, Manuel Serrano-Ruiz^2^, Maurizio Peruzzini^2^, Luigi Ambrosio^1^, Maria Grazia Raucci^1*^**

^1^Institute of Polymers, Composites and Biomaterials – National Research Council (IPCB-CNR), Mostra d’Oltremare pad.20 - Viale J.F. Kennedy 54, 80125 Naples, Italy

^2^Institute of Chemistry of Organometallic Compounds – National Research Council (ICCOM-CNR), via Madonna del Piano 10, 50019 Sesto Fiorentino, Italy

***Corresponding Authors:**

**Dr. Maria Grazia Raucci**

e-mail: mariagrazia.raucci@cnr.it

phone: +39 081 2425945

fax: +39 081 2425932

**Dr. Ines Fasolino**

e-mail: ines.fasolino@cnr.it

phone: +39 081 2425945

fax: +39 081 2425932

**
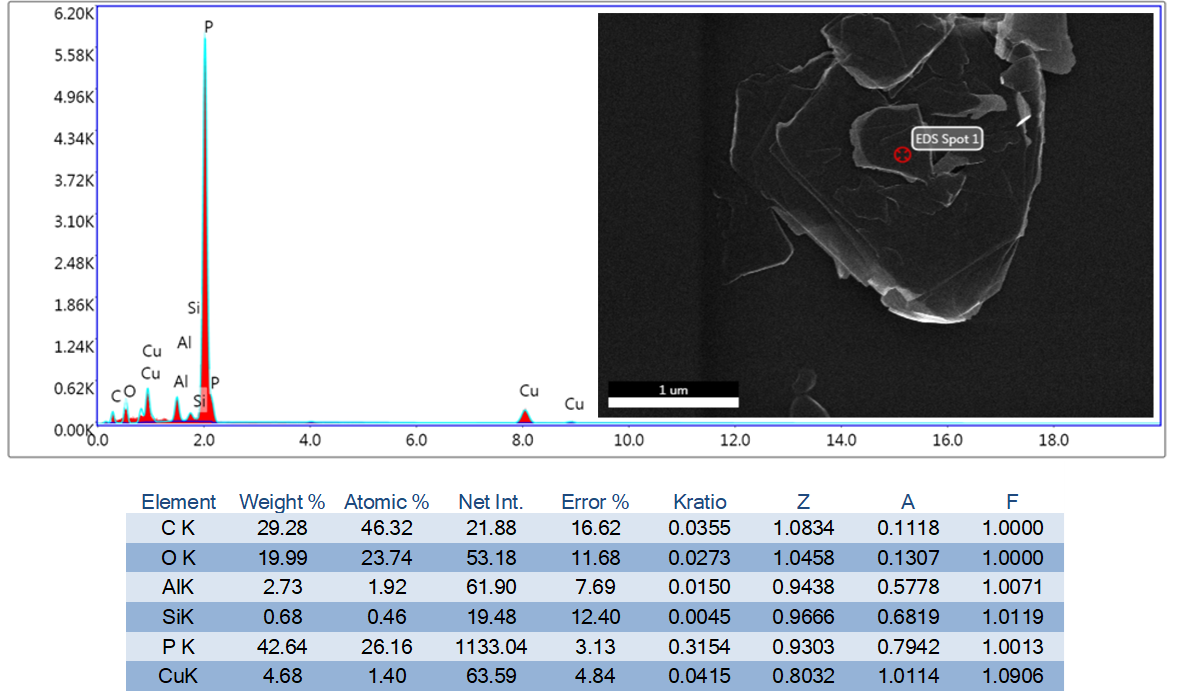
**

**Figure S1.** EDS analysis of the surface of 2D BP shown in the insert. Cu and Al impurities are due to the copper grid where the sample is dropcasted and to the support used to run the measure.


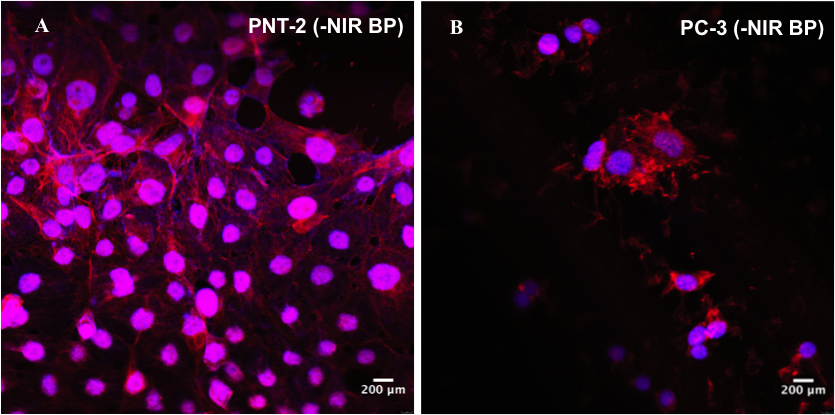


**Figure S2. Effect of non irradiated (-NIR) 2D BP on cell morphology (Rhodamine phalloidin and DAPI staining).**  PNT-2 (A) and PC-3 (B) morphology after 72 hours of exposure to 2D BP (5μg/mL). The images are representative of three experiments.

**
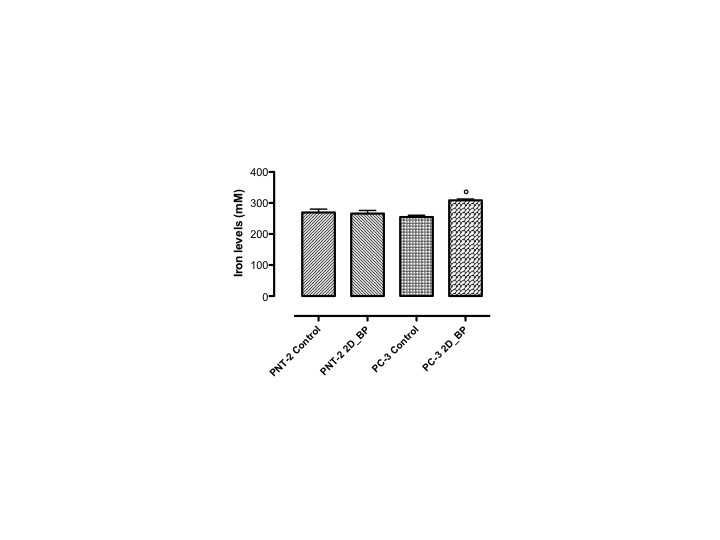
**

**Figure S3. Effect of 2D BP on intracellular Iron amount in PNT-2 and PC-3 cells.** 2D BP does not change Iron levels in PNT-2 cells after 48 hours of exposure but significantly (°p≤0.001) increases Iron amount in PC-3 cells at the same time point. Results are mean ± SEM of 3-4 experiments.


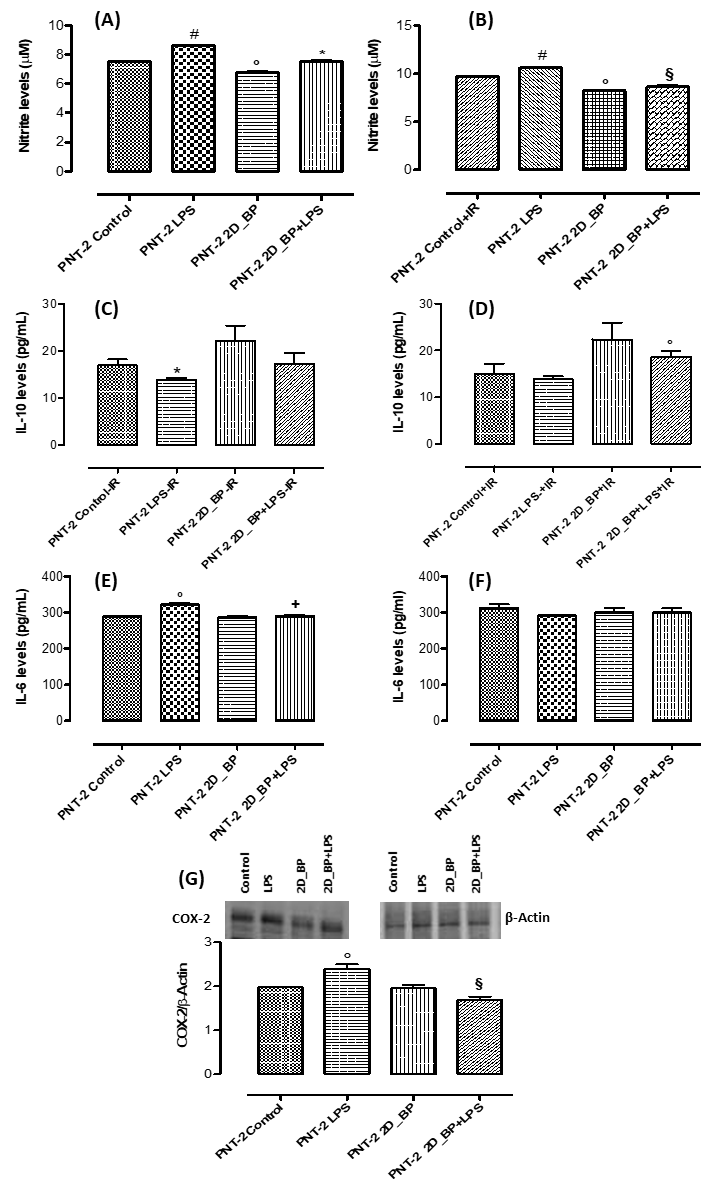


**Figure S4. Effect of irradiated and not irradiated 2D BP on inflammatory response in PNT- 2.** 2D BP without and with irradiation significantly (*p≤0.05, ^§^p≤0.0001) decreases nitrites levels induced by LPS (1 μg/mL) after 72 hours of exposure (^#^p≤0.0001) in PNT-2 cells (A-B). Data on PC-3 are not shown because BP killed cancer cells at this time point. 2D BP without and with irradiation increases IL-10 levels reduced by LPS (1 μg/mL) after 72 hours of exposure (^#^p≤0.05) in PNT-2 cells (C). This effect is more significant (°p≤0.001) in presence of NIR-irradiation (D). 2D BP without irradiation significantly (°p≤0.001) decreases IL-6 (pro-inflammatory cytokine) levels induced by LPS (1 μg/mL) after 72 hours of exposure (+p≤0.001) in PNT-2 cells (E). These results are not significant in presence of NIR-irradiation (F). 2D BP without irradiation significantly ^§^p≤0.0001 reduces COX-2 induced by LPS (1 μg/mL) after 72 hours (°p≤0.001) of exposure in PNT-2 cells (G). Results are mean ± SEM of 3-4 experiments.

**
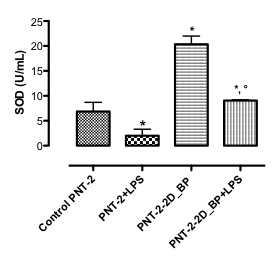
**

**Figure S5. Effect of 2D BP on SOD activity in PNT-2.** Effect of 2D BP on superoxide dismutase (SOD) activity in LPS induced phlogistic stimuli. SOD activities were analysed 3 days after LPS stimulation. 2D BP exposure was performed 24-hours before the inflammatory insult. *p < 0.05 *vs* Control PNT-2 and °p < 0.001 vs PNT-2+LPS. Results are mean ± SEM of 3-4 experiments.
